# Concomitant Use of Steroids and Immunotherapy in a Patient With Paraneoplastic Dermatomyositis and Gastroesophageal Adenocarcinoma

**DOI:** 10.7759/cureus.47628

**Published:** 2023-10-25

**Authors:** Fernando Poli De Frias, Ryan Keith Petit, Carlos Peña, Francesca Polit, Robert Poppiti, Carlos Sesin

**Affiliations:** 1 Department of Internal Medicine, Mount Sinai Medical Center, Miami Beach, USA; 2 Department of Pathology, Mount Sinai Medical Center, Miami Beach, USA; 3 Division of Arthritis and Rheumatology, Mount Sinai Medical Center, Miami Beach, USA

**Keywords:** immune-related adverse effects, oral glucocorticoids, cancer immunotherapy, paraneoplastic dermatomyositis, gastroesophageal adenocarcinoma

## Abstract

Idiopathic inflammatory myopathies (IIMs) are a heterogeneous group of autoimmune pathologies often associated with occult malignancies. Glucocorticoids (GCs) represent the initial therapy to control symptoms and avoid complications. Immune checkpoint inhibitors (ICIs) have shifted the paradigm of cancer treatment. Nivolumab has become the first-line therapy in combination with chemotherapy for untreated, unresectable, non-HER-2-positive advanced gastroesophageal adenocarcinoma. The use of ICIs increases the risk of immune-related adverse events (irAEs), especially in patients with autoimmune diseases, and patients receiving steroids or immunosuppressants might be associated with poorer immunotherapy efficacy. We describe the case of a 49-year-old male who was diagnosed with paraneoplastic dermatomyositis (PDM) and gastroesophageal adenocarcinoma. He was started on prednisone taper, and concomitantly, he was started on chemotherapy with fluorouracil, leucovorin, oxaliplatin, and docetaxel (FLOT), with administration of pegfilgrastim and dexamethasone during each cycle. Additionally, he was started on nivolumab. His course was complicated by worsening episodes of myopathies due to the immunotherapy, requiring adjustments to the prednisone taper. A positron emission tomography (PET) scan and repeat endoscopic ultrasonography with biopsy eight months after therapy initiation showed no major evidence of disease compared to prior. In our case, we exemplified the importance of multidisciplinary management for dosing and tapering of GCs and timing of ICI initiation, and we described the successful response to nivolumab in a patient with autoimmune disease concurrently receiving GCs.

## Introduction

Dermatomyositis (DM) and polymyositis (PM) are autoimmune diseases that form part of a heterogeneous group of pathologies called idiopathic inflammatory myopathies (IIMs). These IIMs are classified based on patterns of presentation, age of onset, immunohistopathologic features, and response to treatment. The goal of treatment is to improve muscle strength, avoid the development of extramuscular complications, and resolve cutaneous manifestations. Glucocorticoids (GCs) are the cornerstone of initial therapy for DM and PM, despite the absence of placebo-controlled trials. The increased risk of malignancy in this population has been widely reported, and many times, this can represent a paraneoplastic manifestation of an underlying occult malignancy [1].

Programmed cell death protein (PD-1) and its ligands PD-L1/PD-L2 are a group of transmembrane proteins widely expressed in immune cells, such as T cells, B cells, macrophages, dendritic cells, and tumor cells. In normal conditions, the interaction of these proteins results in T cell exhaustion, lack of response to stimuli, and altered transcriptional and epigenetic states, required to maintain immunologic homeostasis acting as “natural breaks” that contribute to avoiding autoimmune responses [2]. Immune checkpoint inhibitors (ICIs) are cancer therapies that block this interaction and activate the immune system to fight tumor cells. In the last few decades, antibodies targeting PD-1 or PD-L1 have rapidly become the treatment of choice for multiple solid malignancies [3].

Since ICIs exhibit their anticancer activity through T cell overactivation and, therefore, stimulate the immune system, they can be associated with a wide variety of immune-related adverse events (irAEs), including but not limited to pulmonary, gastrointestinal, dermatological, musculoskeletal, and endocrine systems. Moreover, patients with an already diagnosed autoimmune disease are more susceptible to presenting with an irAE [4].

Glucocorticoids possess well-known potent anti-inflammatory and immunomodulatory properties, mainly through transcriptional repression of cellular proliferation and cytokine production pathways, induction of lymphopenia, and impairment of the T cell response to tumor antigens, and most of the time, GCs represent the main treatment for autoimmune conditions. Theoretically, this suppressive role might counteract the anticancer efficacy of ICIs. However, there is a paucity of clinical data about corticosteroid dosage and tapering, in relation to ICI initiation. We present the case and describe the management and complications of a patient with paraneoplastic dermatomyositis (PDM) as a presenting sign of an occult gastroesophageal junction (GEJ) adenocarcinoma.

## Case presentation

A 49-year-old male with a history of non-Hodgkin lymphoma treated with radiation and chemotherapy 20 years prior and currently in remission, hypothyroidism self-managed with natural over-the-counter supplementation, and former alcohol use presented with six months of progressive night sweats, 20 pounds of weight loss, and intermittent abdominal pain associated with early satiety. Additionally, he complained of diffuse joint pain, generalized fatigue, and muscle weakness, especially related to standing from a sitting position and raising his arms over his head. Initial evaluation showed vital signs within normal ranges. Physical examination was significant for a V-shaped erythematous rash extending across the shoulders, upper back, and neck. Muscle strength testing revealed significant proximal muscle weakness in neck flexors and proximal upper and lower extremities. Lastly, there was mild tenderness with light palpation of the epigastric region.

Laboratory workup was significant for normocytic anemia, normal white blood cell count with neutrophilia, mild thrombocytosis, transaminitis, and elevated inflammatory and myositis-associated markers (Table 1). Antinuclear antibody was positive; however, the myositis-specific panel was negative (Table 2).

**Table 1:** Summary of laboratory workup at initial presentation. Highlighted: abnormal results CBC: complete blood count, WBC: white blood cell, RBC: red blood cell, MCV: mean corpuscular volume, MCH: mean corpuscular hemoglobin, MCHC: mean corpuscular hemoglobin concentration, CMP: comprehensive metabolic panel, BUN: blood urea nitrogen, EGFR: estimated glomerular filtration rate, AST: aspartate aminotransferase, ALT: alanine aminotransferase, CRP: C-reactive protein, ESR: erythrocyte sedimentation rate, LDH: lactate dehydrogenase, CPK: creatine phosphokinase, INR: international normalized ratio, PT: prothrombin time, PTT: partial thromboplastin time, SARS-CoV-2: severe acute respiratory syndrome coronavirus 2, RT-PCR: real-time polymerase chain reaction, NPS: nasopharyngeal swab, ND: not done

Laboratory test (ranges)	Results at initial presentation
CBC	
WBC (4-10.5 × 10^3^/uL^3^)	9.2 × 10^3^/uL^3^
RBC (4.2-5.6 × 10^6^/µL)	4.03 × 10^6^/µL
Hemoglobin (13.3-16.3 g/dL)	10.2 g/dL
Hematocrit (39%-47%)	33.7%
MCV (80-100 µm^3^)	83.6 µm^3^
MCH (27.1-31.1 pg/cell)	25.3 pg/cell
MCHC (32.2-36.5 g/dL)	30.3 g/dL
Platelets (140-400 × 10^3^/µL)	435 × 10^3^/µL
Neutrophils (36%-70%)	88.9%
Lymphocytes (16%-43%)	6.3%
Monocytes (6%-12%)	2.1%
Eosinophils (0%-5%)	1.6%
Basophils (0%-1.2%)	0.2%
Immature granulocytes	0.9
CMP	
Sodium (135-146 mmol/L)	136 mmol/L
Potassium (3.5-5.5 mmol/L)	4.4 mmol/L
Chloride (98-110 mmol/L)	99 mmol/L
CO2 (19-34 mmol/L)	28 mmol/L
BUN (6-20 mg/dL)	13 mg/dL
Creatinine (0.4-1.1 g/day)	0.78 g/day
EGFR (>60 mL/minute/1.75 m^2^)	>90 mL/minute/1.75 m^2^
Glucose (65-99 mg/dL)	108 mg/dL
Calcium (8.6-10.3 mg/dL)	9 mg/dL
Alkaline phosphatase (40-130 units/L)	141 units/L
Total bilirubin (0-1.2 mg/dL)	0.5 mg/dL
Total protein (6.1-8.1 g/dL)	6.9 g/dL
Albumin (3.5-5.2 g/dL)	3.5 g/dL
AST (10-40 units/L)	273 units/L
ALT (10-55 units/L)	249 units/L
Inflammatory markers	
CRP (<8 mg/L)	64.1 mg/L
ESR (<15 mm/hour)	99 mm/hour
Ferritin (38-380 mg/dL)	2,236 mg/dL
LDH (<220 U/L)	414 U/L
CPK (44-196 U/L)	2,595 U/L
Aldolase (<8.1 U/L)	20.6 U/L
Coagulation	
INR (<1.1)	1.2
PT (9-11.5 seconds)	11.6 seconds
PTT (23-32 seconds)	28 seconds
D-Dimer (<0.5 mcg/mL FEU)	2.14 mcg/mL FEU
Microbiology	
Blood culture	Negative
Urine culture	Negative
SARS-CoV-2/RT-PCR NPS	Negative
HIV ½ AG/AB, 4^th^ generation	Non-reactive
Rapid plasma reagin	Negative
Acute hepatitis serologies	Negative

**Table 2:** Autoimmunity markers and myositis-specific antibodies. Highlighted: abnormal results AB: antibodies, ANA: antinuclear antibodies, AMA: antimitochondrial antibody, anti-dsDNA ab: anti-double-stranded DNA, SM: anti-Smith, RNP: ribonucleoprotein, SRP: signal recognition particle, TIF1γ: transcriptional intermediary factor 1 gamma, HMGCR: 3-hydroxy-3-methyl-glutaryl-coenzyme A reductase

Laboratory test	Results at initial presentation
Autoimmunity markers	
ANA titer (<1:40)	1:1280
ANA pattern	Cytoplasmic, reticular/AMA
Anti-dsDNA ab (<30 IU/mL)	12 IU/mL
Anti-SM Ab (<1 AI)	<1 AI
Anti-SM/RNP Ab (<1 AI)	<1 AI
Anti-JO-1 AB (<11 SI)	<11 SI
Anti-PL-7 AB (<11 SI)	<11 SI
Anti-PL-12 AB (<11 SI)	<11 SI
Anti-EJ AB (<11 SI)	<11 SI
Anti-OJ AB (<11 SI)	<11 SI
Anti-SRP AB (<11 SI)	<11 SI
Anti-MI-2 ALPHA AB (<11 SI)	<11 SI
Anti-MI-2 BETA AB (<11 SI)	<11 SI
Anti-MD5 AB (<11 SI)	<11 SI
Anti-TIF1γ AB (<11 SI)	<11 SI
Anti-NXP-2 AB (<11 SI)	<11 SI
Anti-HMGCR AB (<20 CU)	<11 CU

**Figure 1:**
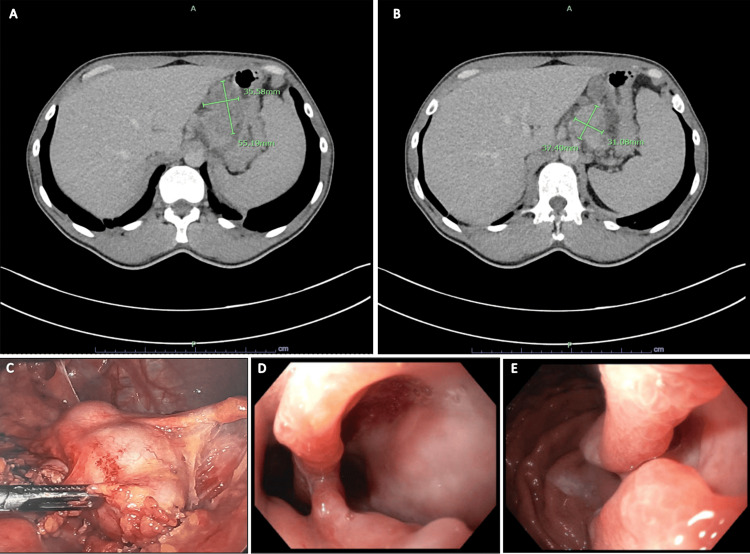
Initial evaluation with CT scan of the abdomen with IV contrast and endoscopic findings. (A) Soft tissue is seen along the gastro-hepatic ligament, which effaces along the subserosa surface of the lesser curvature of the stomach in the gastric body with a conglomerate measurement of approximately 3.6 × 5.5 cm. (B) Bulky gastro-hepatic lymphadenopathy measuring up to 3.1 × 3.7 cm. (C) Exploratory laparoscopy revealed bulky lymphadenopathy in the celiac trunk along the lesser curvature of the stomach compressing the left gastric artery. (D and E) Endoscopic findings revealed a fungating and ulcerating mass with bleeding upon contact at the GEJ, extending 40-45 cm from the incisors. The Z-line is located at 41 cm. The mass was non-obstructive and partially circumferential, involving one-third of the lumen circumference. CT: computed tomography, GEJ: gastroesophageal junction

**Figure 2:**
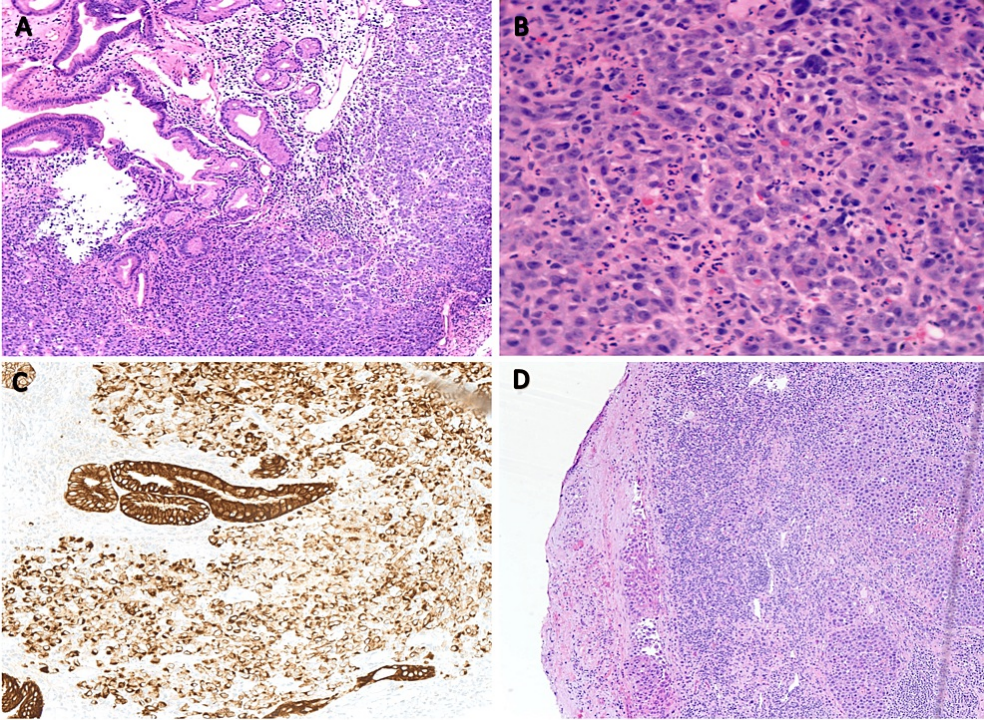
Pathology findings of the gastric mass and perigastric lymph node. (A) Foveolar epithelium and a dense infiltrate of atypical cells with no recognizable glands, representing a malignant neoplasm (50×). (B) Poorly cohesive plasmacytoid infiltrate with pleomorphism and scattered atypical mitoses in a background of acute inflammation, representing a poorly differentiated adenocarcinoma (400×). (C) Positive CAM 5.2 stain, supporting the diagnosis of an epithelial neoplasm (100×). (D) Excisional biopsy of bulky gastro-hepatic lymphadenopathy showing metastatic carcinoma (50×). CAM: cell adhesion molecule

The patient underwent abdominal computed tomography (CT) scan with intravenous contrast, which showed an ill-defined mass in the gastric body with bulky lymphadenopathy along the gastro-hepatic ligament. Upper endoscopic ultrasound (EUS) demonstrated a circumferential, non-obstructive, fungated, and ulcerating mass 4 cm away from the gastroesophageal junction (GEJ) with bleeding upon contact (Figure 1). The endo sonogram showed irregular outer margins with evidence suggesting invasion into the serosa along the lesser curvature of the stomach. Additionally, three malignant lymph nodes were visualized in the gastro-hepatic ligament with clinical T3N2 staging, Siewert 3. He underwent an exploratory laparoscopy that revealed bulky lymphadenopathy in the celiac trunk along the lesser curvature of the stomach, and an excisional biopsy of one lymph node was obtained (Figure 1C). Gastroesophageal mass and lymph node pathology demonstrated poorly differentiated adenocarcinoma (Figure 2). Circulating tumor DNA (ctDNA) testing was high for microsatellite instability-high (MSI-High), and tumor mutational burden (TMB) was 65. The combined positive score (CPS) was calculated to be >40 by immunohistochemistry (IHC), making the patient an excellent candidate for ICI therapy with a PD-L1 inhibitor.

He was diagnosed with paraneoplastic dermatomyositis given his European League Against Rheumatism/American College of Rheumatology (EULAR/ACR) aggregate score of >7.5 corresponding to “definite IIM.” He was started on prednisone 60 mg daily for three weeks until clinical improvement was achieved, followed by weekly tapering by 10 mg. Concomitantly, he was started on chemotherapy with fluorouracil, leucovorin, oxaliplatin, and docetaxel (FLOT), with concomitant administration of pegfilgrastim and dexamethasone during each cycle. Interestingly, after each chemotherapy cycle, the patient felt significant improvement in his muscular weakness given the dexamethasone infusion. After the initiation of steroids and chemotherapy, the patient had improvement in his generalized weakness and fatigue. Muscle strength testing, inflammatory markers, and muscle enzymes markedly improved over the course of three weeks (Table 3). 

**Table 3:** Laboratory monitoring in relation to treatment cycle and therapies received. Highlighted: abnormal results ND: not done, AST: aspartate aminotransferase, ALT: alanine aminotransferase, CPK: creatine phosphokinase, LDH: lactate dehydrogenase, CRP: C-reactive protein, ESR: erythrocyte sedimentation rate, CA-125: cancer antigen-125, TSH: thyroid-stimulating hormone, T4: thyroxine, FLOT+: fluorouracil (2.6 g/m^2^), leucovorin (200 mg/m^2^), oxaliplatin (85 mg/m^2^), docetaxel (50 mg/m^2^), dexamethasone (10 mg IV), and pegfilgrastim (6 mg subcutaneous), Niv: nivolumab (480 mg IV infusion), Pred: prednisone (oral daily)

	Treatments	AST (10-40 units/L)	ALT (10-55 units/L)	CPK (44-196 U/L)	LDH (<220 U/L)	CRP (<8 mg/L)	ESR (<15 mm/hour)	CA-125 (0-35 U/mL)	TSH (0.4-4.50 ml/UL)	T4 free (0.8-1.8 ng/dL)
Initial laboratory test	Pred 60 mg	273 units/L	249 units/L	2,595 U/L	414 U/L	64.1 mg/L	99 mm/hour	70 U/mL	8.77 ml/UL	2.1 ng/dL
Cycle 1	FLOT+/Pred 50 mg	231 units/L	174 units/L	455 U/L	ND	ND	ND	ND	ND	ND
Cycle 2	FLOT+/Pred 40 mg	85 units/L	161 units/L	507 U/L	284 U/L	14.8 mg/L	ND	28 U/mL	ND	ND
Cycle 3	FLOT+/Niv/Pred 20 mg	67 units/L	60 units/L	401 U/L	ND	ND	ND	ND	ND	ND
Cycle 4	FLOT+/Niv/Pred 20 mg	63 units/L	61 units/L	747 U/L	1,164 U/L	5.3 mg/L	9 mm/hour	24 U/mL	ND	ND
Cycle 5	FLOT+/Niv/Pred 10 mg	75 units/L	59 units/L	732 U/L	ND	ND	ND	ND	8.56 ml/UL	1.3 ng/dL
Cycle 6	FLOT+/Niv/Pred 10 mg	85 units/L	69 units/L	453 U/L	130 U/L	2.6 mg/L	ND	25 U/mL	ND	ND
Cycle 7	Niv/Pred 10 mg	39 units/L	31 units/L	279 U/L	ND	ND	ND	ND	ND	ND
Cycle 8	Niv/Pred 7.5 mg	30 units/L	20 units/L	191 U/L	ND	0.5 mg/L	2 mm/hour	17 U/mL	5.61 ml/UL	0.9 ng/dL
Cycle 9	Niv/Pred 5 mg	23 units/L	19 units/L	187 U/L	ND	ND	ND	ND	ND	ND
Cycle 10	Niv	28 units/L	21 units/L	ND	ND	ND	ND	16 U/mL	13.17 ml/UL	0.8 ng/dL
Cycle 11	Niv	24 units/L	16 units/L	ND	ND	ND	ND	ND	ND	ND
Cycle 12	Niv	25 units/L	21 units/L	ND	ND	0.6 mg/L	2 mm/hour	14 U/mL	ND	ND
Cycle 13	Niv	30 units/L	26 units/L	153	ND	ND	ND	ND	11.53 ml/UL	0.9 ng/dL
Cycle 14	Niv	23 units/L	21 units/L	2,426 U/L	ND	ND	ND	15 U/mL	10.23 ml/UL	0.8 ng/dL
Cycle 15	Niv	51 units/L	26 units/L	387 U/L	ND	ND	ND	ND	14.63 ml/UL	0.8 ng/dL
Cycle 16	Niv	24 units/L	19 units/L	143 U/L	ND	ND	2 mm/hour	ND	14.06 ml/UL	0.9 ng/dL
Cycle 17	Niv	24 units/L	20 units/L	151 U/L	ND	ND	ND	ND	17.84 ml/UL	0.9 ng/dL
Cycle 18	Niv	23 units/L	17 units/L	415 U/L	ND	ND	ND	ND	ND	ND

A multidisciplinary decision-making process involving rheumatology and medical oncology concluded that the benefits of immunotherapy outweighed the risk of worsening AID; therefore, the patient was started on concomitant immunotherapy with nivolumab, which was initiated with the third cycle of chemotherapy. Additionally, it was concluded that steroids needed to be tapered to at least <10 mg while on nivolumab to avoid any potential loss of efficacy.

After the third cycle that included nivolumab, the patient re-experienced proximal muscular weakness, and mild myalgias and arthralgias, with an uptrend of aspartate aminotransferase (AST), creatine phosphokinase (CPK), and lactate dehydrogenase (LDH) over the course of the next weeks, requiring holding further tapering of steroids to control symptoms (Table 3). The patient underwent a total of six cycles of FLOT, followed by continued immunotherapy with nivolumab alone, every four weeks. Myopathy symptoms persisted, and the tapering was restarted after cycle 7 until discontinuing steroids in cycle 9.

**Figure 3:**
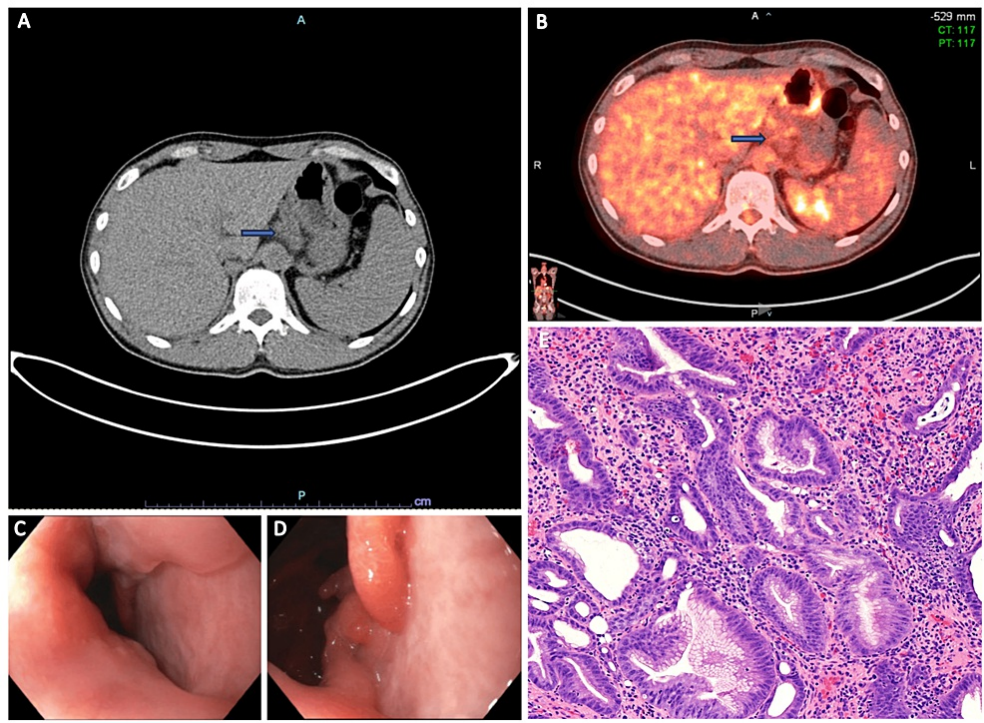
Restaging studies eight months after the start of therapy. (A and B) PET/CT scan with IV F-18 FDG showed heterogeneous soft tissue infiltration along the gastro-hepatic ligament with persistent nodularity measuring up to 2.3 × 2.6 cm, with corresponding mild FDG uptake (SUV max: 2.7) (arrow). (C and D) Upper endoscopy showed an ulcer with elevated borders in the GEJ involving less than one-fourth of the lumen circumference, with no bleeding upon contact. (E) Biopsy of the ulcer at the GEJ showed gastric mucosa with regenerative changes and acute and chronic inflammation of the lamina propria; no residual atypical proliferation of cells was present. PET: positron emission tomography, CT: computed tomography, FDG: fluorodeoxyglucose, SUV max: maximum standard unit value, GEJ: gastroesophageal junction

A positron emission tomography (PET) scan eight months after therapy initiation showed no evidence of prior ill-defined mass in the gastric body and a decrease in size of gastro-hepatic lymphadenopathy, with mild fluorodeoxyglucose (FDG) uptake, suggesting no major evidence of disease. An esophagogastroduodenoscopy and endoscopic ultrasonography (EUS) performed at this time showed a GEJ circumferential ulcer with no mass effect compared to before, and five malignant-appearing lymph nodes at the gastro-hepatic ligament reduced in size. However, there was no evidence of disease on the gastric mucosal biopsy of the ulcerating lesion nor fine needle aspiration (FNA) of the lymph nodes (Figure 3).

The patient has continued to tolerate nivolumab therapy, currently on cycle 18. He re-experienced proximal myalgias on cycle 18 with uptrending CPK and worsening thyroid-stimulating hormone (TSH); however, these symptoms self-resolved (Table 3). He was prescribed levothyroxine 25 mcg due to uptrending TSH and his prior history of hypothyroidism. He has gained weight and resumed physical activities without any recurrence of rashes, myalgias, arthralgias, or fatigue.

## Discussion

IIMs have been revised and reclassified in multiple studies over the years. However, the EULAR/ACR classification criteria correctly classified most patients with all IIM sub-diagnoses using a score-based analysis with a subsequent decision tree analysis, with high diagnostic performance in comparison with prior studies. In the case of our patient, he presented initially with a photo-distributed poikiloderma on the neck, back, shoulders (shawl sign), and anterior chest (V sign) that self-resolved over time, which can be seen in some subtypes of DM called amyopathic. Subsequently, he developed a very common pattern of generalized muscle pain and proximal muscle weakness in the upper and lower extremities, with elevated muscle enzymes and inflammatory markers. His EULAR/ACR aggregate score was >7.5, corresponding to “definite IIM,” and given the skin findings and proximal muscle myopathy, he was diagnosed with DM. In the absence of autoantibodies, it was thought to be paraneoplastic in nature [5]. Myositis-specific antibodies (MSA), such as Jo-1, aminoacyl tRNA synthetases (ARS), anti-Mi-2, and anti-signal recognition particle (SRP), among others, are associated with different clinical subtypes [6]. Anti-TIF1-γ and anti-NXP-2 have been linked to the presence of neoplasia [7]. However, their absence does not rule out disease, and they are not required to establish a diagnosis [8].

PD-/PD-L1 inhibitors have shifted the paradigm of cancer treatment. Nivolumab has become the first-line therapy in combination with chemotherapy for untreated, unresectable, non-HER-2-positive advanced gastric, GEJ, or esophageal adenocarcinoma [9]. Characteristics and biomarkers in successful, long-term survival cases have not been identified; however, MSI and CPS are good predictors of therapeutic efficacy, which, in our patient, were positive [10]. Despite the residual gastro-hepatic ligament lymphadenopathy, which was decreased in size compared to prior, our patient achieved a significant response after six months of treatment with nivolumab, evidenced by complete resolution of the mass seen previously on the EUS, repeated biopsy without findings of disease on specimens collected, and PET/CT without major metabolic activity.

Despite great efficacy, ICIs are not exempt from undesirable effects, especially irAEs, which have been reported recently in cancer patients, and drugs such as ipilimumab, nivolumab, and pembrolizumab are responsible for almost 60% of the reported cases. Additionally, most cases with preexisting autoimmune disease (PAD) were associated with a modest increase in hospitalizations related to an irAE and corticosteroid use [11]. However, most of the manifestations were mild to moderate and did not require ICI discontinuation, and higher grades of irAEs have been seen with CTLA-4 inhibitors as this blockade might induce a greater magnitude of T cell proliferation in comparison to PD-1 inhibitors [12].

Musculoskeletal manifestations have been reported in individuals receiving ICIs, including myalgias and myositis, with a frequency of 4% and 0.6%, respectively; hypothyroidism is the most common endocrinopathy seen in 6%-22% of the cases [12]. Other studies have further characterized ICI-associated inflammatory myopathies, reporting a mean time of onset of 25 days after ICI initiation [13]. Despite gentle and patient-tolerated tapering of GCs to initiate ICIs, our patient developed a recurrence of muscle weakness and myalgias with an increase in muscle enzyme and inflammatory markers after starting nivolumab, which was thought to be an exacerbation of his PDM secondary to the recent start of immunotherapy. However, symptomatology was controlled by holding the steroid taper. Additionally, he developed worsening hypothyroidism later on therapy, with transient elevation of CPK, without any clinical manifestation. As per current American Society of Clinical Oncology (ASCO) guidelines, nivolumab was continued as these were grade 1-2 irAEs [14].

However, a multicenter retrospective study concluded that the development of irAEs was among the factors associated with better outcomes in immunotherapy in metastatic gastric adenocarcinoma and various types of cancers. As ICIs reactivate suppressed T cells, irAEs are likely the manifestation of the immune cell activation and, therefore, are related to the antitumor effect of nivolumab. Furthermore, T cells enhance the effect of treatment with the PD-1 antibody, which may in turn induce autoantibodies via B cells, thereby promoting the development of irAEs [15].

Whether the use of steroids and/or immunosuppressants to treat irAEs could reduce the efficacy of ICIs has been evaluated in several studies. Preclinical data in mouse models have shown that the activation of GC receptor deeply inhibited pro-inflammatory cytokine production, induced multiple checkpoint inhibitor expression in CD8+ T cells, and was associated with poor response to immune checkpoint blockade [16]. In the setting of advanced non-small cell lung cancer, baseline corticosteroid treatment with ≥10 mg of prednisone equivalent daily at the start of PD-1 blockade or exposure to glucocorticoids during the first nivolumab cycle was associated with poorer outcomes [17]. Similar findings were described in a cohort of patients with glioblastoma, where concurrent dexamethasone administration, whether <2 mg or >2 mg, at the time of the initiation of anti-PD-1 therapy, was the single strongest predictor of poor survival [18]. Nevertheless, current data is conflicting, and recent literature reviews and studies on metastatic renal cell carcinoma and melanoma may not necessarily lead to poorer clinical outcomes, although it was not possible to define a dose or exposure threshold beyond which corticosteroids might be associated with poorer immunotherapy efficacy [19]. However, many of these studies use GCs to treat irAEs in patients already started in ICIs, for which exposure to GCs before the introduction or early after ICI initiation appears to be more often associated with poorer response. Several clinical studies have highlighted a negative association between systemic corticosteroid use and overall survival (OS) and tumor response in patients receiving glucocorticoids prior to the initiation of the ICIs [20].

## Conclusions

In our case, we exemplified the importance of a comprehensive assessment given the relationship between IIMs and occult malignancies. Although published available data suggest that ongoing GC therapy at the time of ICI initiation is associated with poorer response, in our case, it did not affect the clinical efficacy of the immunotherapy. However, the dose was tapered at its minimum as tolerated per patient, with proper multidisciplinary management between oncology and rheumatology. High MSI and CPS are good predictors of therapeutic efficacy, and our patient had a successful and rapid response to nivolumab; however, prognostic factors to ICIs in gastric adenocarcinoma need further investigation. The development of irAEs is described among the factors related to better outcomes. irAEs are common in patients receiving ICI therapy, especially in patients with known autoimmune diseases, and despite the majority of these being reported to be low grade, discontinuation of therapy should be based on current publicly available guidelines and at the discretion of the treating team.
